# Insulin-degrading enzyme is not secreted from cultured cells

**DOI:** 10.1038/s41598-018-20597-6

**Published:** 2018-02-05

**Authors:** Eun Suk Song, David W. Rodgers, Louis B. Hersh

**Affiliations:** Department of Molecular and Cellular Biochemistry and the Center for Structural Biology, University of Kentucky, Lexington, KY 40536 USA

## Abstract

Insulin-degrading enzyme (IDE) functions in the catabolism of bioactive peptides. Established roles include degrading insulin and the amyloid beta peptide (Aβ), linking it to diabetes and Alzheimer’s disease. IDE is primarily located in the cytosol, and a longstanding question is how it gains access to its peptide substrates. Reports suggest that IDE secreted by an unconventional pathway participates in extracellular hydrolysis of insulin and Aβ. We find that IDE release from cultured HEK-293 or BV-2 cells represents only ~1% of total cellular IDE, far less than has been reported previously. Importantly, lactate dehydrogenase (LDH) and other cytosolic enzymes are released at the same relative level, indicating that extracellular IDE results from a loss of cell integrity, not secretion. Lovastatin increases IDE release from BV-2 cells as reported, but this release is mirrored by LDH release. Cell viability assays indicate lovastatin causes a loss of cell integrity, explaining its effect on IDE release. IDE is present in an exosome-enriched fraction from BV-2 cell conditioned media, however it represents only ~0.01% of the total cellular enzyme and is unlikely to be a significant source of IDE. These results call into question the secretion of IDE and its importance in extracellular peptide degradation.

## Introduction

Insulin-degrading enzyme (IDE) is a zinc metallopeptidase that degrades a number of physiological peptides, the best documented *in vivo* substrates being insulin and amyloid β-peptide (Aβ). IDE is composed of four structurally similar domains folded in a clamshell-like shape, and it functions primarily as a homodimer. With some of its smaller peptide substrates and with amyloid β-peptide, IDE exhibits allosteric kinetic behavior^1^. IDE is mostly localized to the cytosol of the cell, but has also been observed in mitochondria^2^, peroxisomes^3^ and endosomes^4–7^. There have been a number of studies reporting the secretion of IDE from cells^6,8–13^ and this secreted form of IDE has been suggested to play an important role in degrading insulin and Aβ. Investigations into the mechanism of IDE secretion by Zhao *et al*.^12^ found that classical inhibitors of protein secretion, namely brefeldin A, monensin, and nocodazole, were ineffective in blocking IDE secretion. The lack of inhibition of IDE secretion by brefeldin A and monensin was confirmed by Bulloj *et al*.^6^. This led to the suggestion that IDE was secreted from cells via an unconventional mechanism, similar to that proposed for a number of other proteins^14^. A C-terminal SlyX motif in IDE was proposed to act as a secretion signal^15^ but was subsequently found to increase IDE lifetime by decreasing lysosomal degradation rather than directly affecting secretion^9^. It has further been reported that IDE is secreted in exosomes from astrocytes^6,16^ by an autophagy-dependent secretory pathway^9,10^.

Secretion of IDE from cells and its presence in the extracellular space would have major implications in its physiological function. As noted, IDE has been implicated as one of the major enzymes involved in Aβ degradation, and genetic studies link IDE to late onset Alzheimer’s disease^17,18^. The report of IDE secretion has led to the postulate that IDE degrades at least some Aβ extracellularly. In addition, IDE is a major insulin-metabolizing enzyme thought to degrade insulin intracellularly within endosomes, however it has been suggested that at least a fraction of insulin metabolism by IDE may occur extracellularly^19^.

We recently uncovered a mechanism for the localization of IDE to endosomes via interaction with phosphoinositides^7^. During the course of these studies we asked whether the phosphatidylinositol dependent trafficking of IDE to endosomes or other intracellular compartments could play a role in IDE secretion. We thus initiated an investigation of IDE secretion from a number of cell lines previously reported to secrete IDE into the media. To our surprise we could not confirm any specific secretion of IDE, finding that lactate dehydrogenase (LDH) and other cytosolic enzymes were also released at the same relative levels as IDE. Since LDH in the media of cultured cells is considered a marker for cell lysis, our results suggest that IDE found in conditioned media results from loss of cell integrity rather than specific secretion, at least in the cell lines studied.

## Results

### IDE Release from HEK Cells

**Figure 1:**
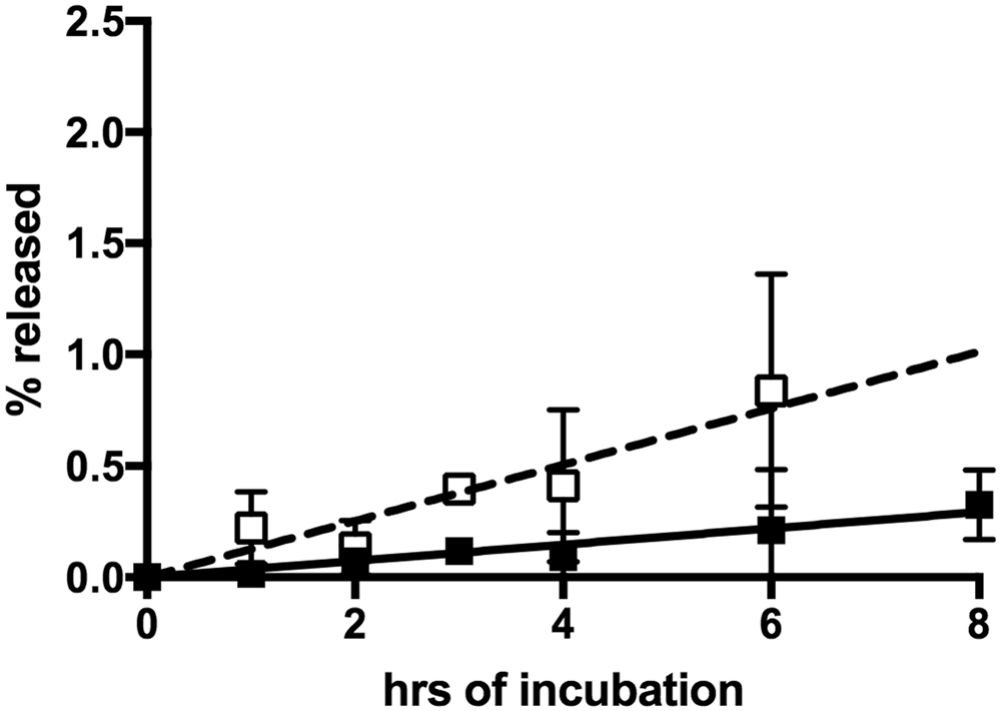
Release of IDE and LDH from HEK-293 cells. HEK-293 cells were cultured in DMEM media with 10% FBS. At the time periods indicated aliquots were taken and used for Western blot analysis for IDE (■) or LDH (◻). The % released is based on the Western blot signal in the media relative to the total Western blot signal (media plus cell lysate). Indicated points are the mean of results from five independent experiments with the error bars represent +/− the standard deviation of the measurements. The lines show linear regression fits to the IDE values (solid line) or LDH values (dashed line).

To test for the secretion of endogenous IDE from HEK-293 cells we grew these cells overnight in DMEM media containing 10% FBS, taking precautions to avoid any manipulations of the cells that might disrupt cellular function. The media was then carefully changed to initiate the experiment. Aliquots of the conditioned media were taken at fixed time points, and Western blot analysis was used to assess the IDE content of the media without further manipulation. Lactate dehydrogenase (LDH) content in the media was used as a control for a cytosolic non-secreted protein. The result of this analysis showed a small amount of IDE (generally less than 1% of the total cellular enzyme) detectable in the media over a 8 h incubation period, and this amount was essentially the same as the percentage of LDH found in the media (Fig. 1 and Supplementary Fig. S1). Similar results were obtained if separate cultures were used for each time point, but as expected there was more variability in the data. However, again 1% or less IDE along with an equivalent amount of LDH was found in the conditioned media. This led us to conclude that the IDE found in conditioned media is likely not a result of specific secretion but rather is attributable to a small percentage of cells that had lost membrane integrity during the incubation period. We found no significant effect of adding a protease inhibitor cocktail containing AEBSF, aprotinin, bestatin, E-64, leupeptin and pepstatin A to the media during the course of the experiment, indicating that enzyme release was not underestimated due to proteolytic degradation (Supplementary Fig. S9, upper panel). In addition, recombinant rat IDE added to conditioned media from an HEK-293 cell culture did not degrade over the course of an 8 hr incubation (Supplementary Fig. S9, lower panel). A trypan blue exclusion assay failed to detect an increase in compromised cells over the course of the experiment, typically giving ~95% live cells at both the beginning and end of the incubation. However, we would not expect this assay to detect the ~1% change in cell integrity indicated by the magnitude of the observed release of IDE and LDH.

### Release from BV-2 Cells

**Figure 2:**
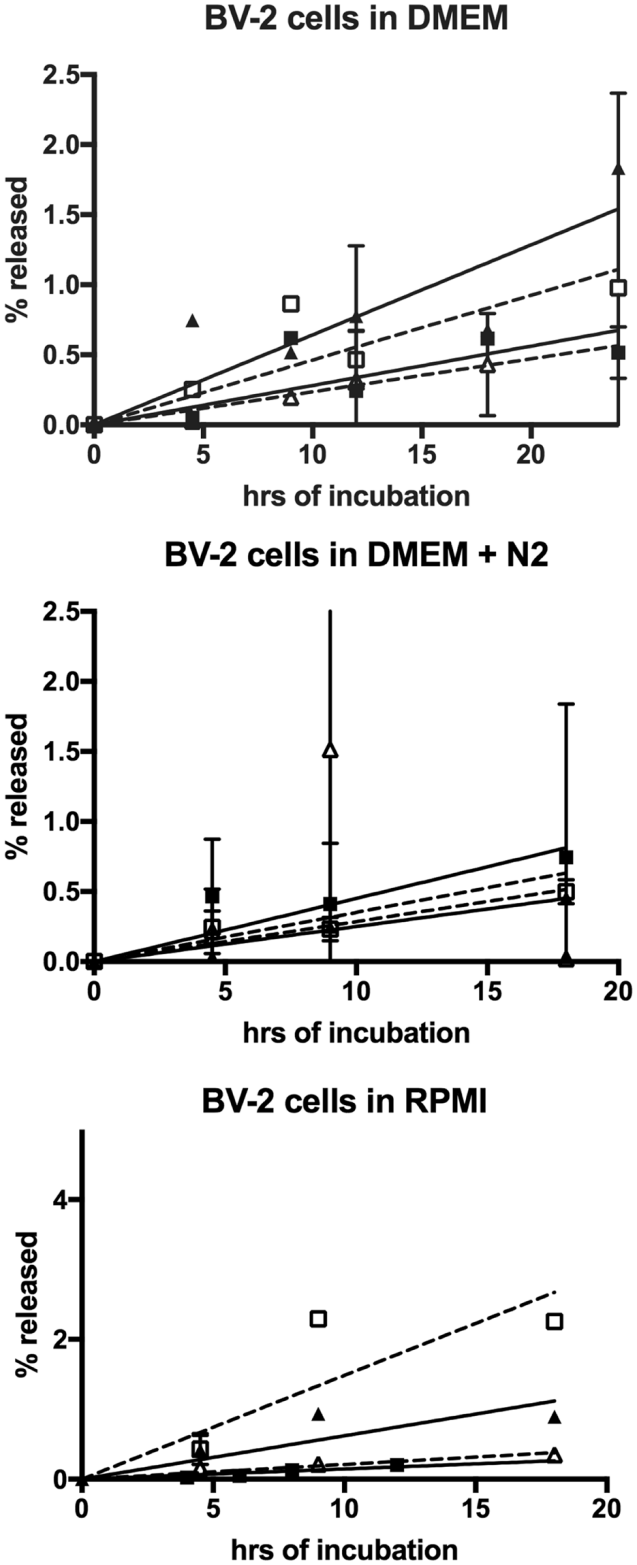
Release of IDE and LDH into media from BV-2 cells under various growth conditions reported in the literature. BV-2 cells were cultured in DMEM media (top panel), DMEM media with N2 supplement (middle panel), or RPMI media (lower panel) in the presence or absence of 10% FBS. At the time periods indicated aliquots were taken and used for Western blot analysis for IDE (+FBS ■, −FBS ▲, solid lines) or LDH (+FBS ☐, −FBS△, dashed lines). The % release is based on the Western blot signal in the media relative to the total Western blot signal (media plus cell lysate). The results are the average values from 2–5 independent trials for each condition except IDE in DMEM + N2 without serum and IDE in RPMI with serum, which are the results from a single series of measurements. Error bars indicate +/− the standard deviation of the of the averaged values.

Since IDE secretion from BV-2 cells has been reported by several investigators^11,16,20^, we next looked at IDE release from these cells under various experimental conditions in which secretion has been reported. In the DMEM media containing 10% fetal bovine serum (FBS) used by Tamboli *et al*.^16^, we found about 1% of total cellular IDE released into the media after 24 h (Fig. 2 top panel and Supplementary Fig. S2). As seen with HEK cells, the percentage of LDH in the conditioned media was comparable to the percentage of released IDE. The scatter in the data is the result of combining data from different experiments as well as the small amount of IDE and LDH released. In the absence of serum, similar amounts of IDE and LDH were released.

In DMEM + N2 supplement media, we found that BV-2 cells grown in the presence or absence of 10% FBS released less than 1% of total cellular IDE after 18 h, with comparable amounts of total cellular LDH released at that time point (Fig. 2 middle panel and Supplementary Fig. S3). Essentially the same result was obtained when BV-2 cells were cultured in RPMI media as used in Qiu *et al*.^11^ (Fig. 2 bottom panel and Supplementary Fig. S4), where IDE release of less than 1% at 18 h was accompanied by a similar release of LDH. Unfortunately, we cannot compare these amounts to those observed in the study of Qiu *et al*.^11^ since the percentage of cellular IDE secreted into the media by BV-2 cells was not determined in that study.

In measuring BV-2 cell integrity, no differences were observed in the number of trypan blue positive cells between the zero and 18 or 24 h. time points in the presence or absence of FBS. However, we also used a more sensitive MTS (3-(4,5-dimethylthiazol-2-yl)-5-(3-carboxymethoxyphenyl)-2-(4-sulfophenyl)-2Htetrazolium) dye reduction assay^21^ to test for metabolically active cells as another way to monitor cell viability. In this assay a reduction in color production indicates a decrease in cell metabolic capacity, which is usually interpreted as a loss in cell viability. The MTS assay showed an increase of 15–17% in color production between the 0 and 18 h time points in the presence or absence of FBS, consistent with the expected growth of the BV-2 cells. Like the trypan blue assay, this result suggests the loss of cell viability is low, which matches the small percentage of IDE and LDH release.

**Figure 3:**
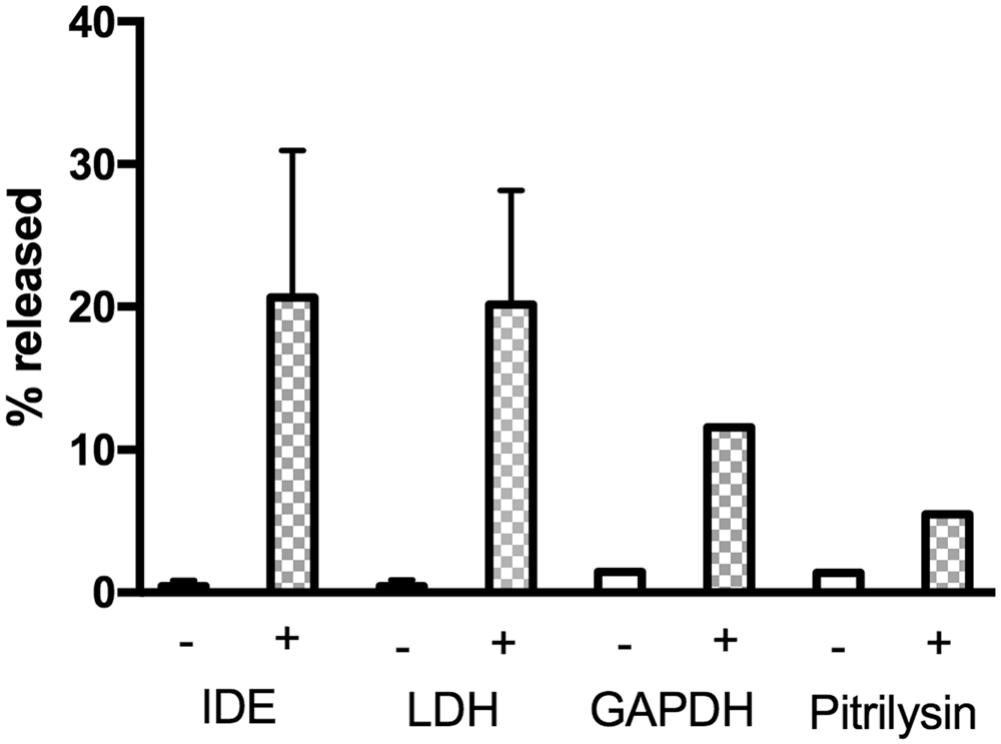
Effect of lovastatin on the release of IDE and other proteins from BV-2 cells cultured in RPMI media. BV-2 cells were cultured in DMEM media for 24 h in the presence (+) or absence (−) of 5 µM lovastatin. The conditioned media were collected and used for measuring IDE, LDH, GAPDH, and pitrilysin by Western blot analysis. Cells were lysed on the plate and the lysate used for measuring the amount of intracellular proteins. The % release is based on the Western blot signal in the media relative to the total Western blot signal (media plus cell lysate). Values for IDE and LDH are the means of three independent trials, and values for GAPDH and Pitrilysin are from single trials. Error bars indicate +/−  the standard deviation of the averaged values.

There have been several reports that statins induce the secretion of IDE from cultured cells, including BV-2 cells^10,16^. Thus we looked at the ability of lovastatin to increase IDE release. Lovastatin did in fact increase IDE release after 24 h, however LDH release was increased to the same extent (Fig. 3 and Supplementary Fig. S5). In this case, the amount of released IDE increased to ~20% of the total cellular enzyme. However, as in other experiments, LDH release mirrored IDE release, suggested that lovastatin affects BV-2 cells by inducing cell dysfunction rather than specifically increasing IDE secretion. Lovastatin also caused an increased release of two other measured cytosolic markers, glyceraldehyde dehydrogenase (GAPDH) and the IDE-related peptidase pitrilysin, further supporting the conclusion that lovastatin-induced IDE release is not a specific effect but rather a more general phenomenon due to a deleterious effect on cell viability. In this case the trypan blue exclusion assay showed a trend toward lovastatin inducing cell death, but did not reach statistical significance (p = 0.18). On the other hand, the MTS dye reduction assay showed a significant effect of lovastatin after 24 h (p = 0.003). In the absence of lovastatin, the MTS assay showed an 11% increase in color production, indicative of cell growth. In contrast in the presence of lovastatin the MTS assay showed a 5% decrease in color production indicative of cell dysfunction. The overall change in color production with and without the statin was 16%, which is consistent with the 5–20% release of IDE, LDH, GAPDH and pitrilysin shown in Fig. 3. Together these assays provide evidence that lovastatin reduces cell integrity, leading to nonspecific release of IDE and other proteins.

**Figure 4:**
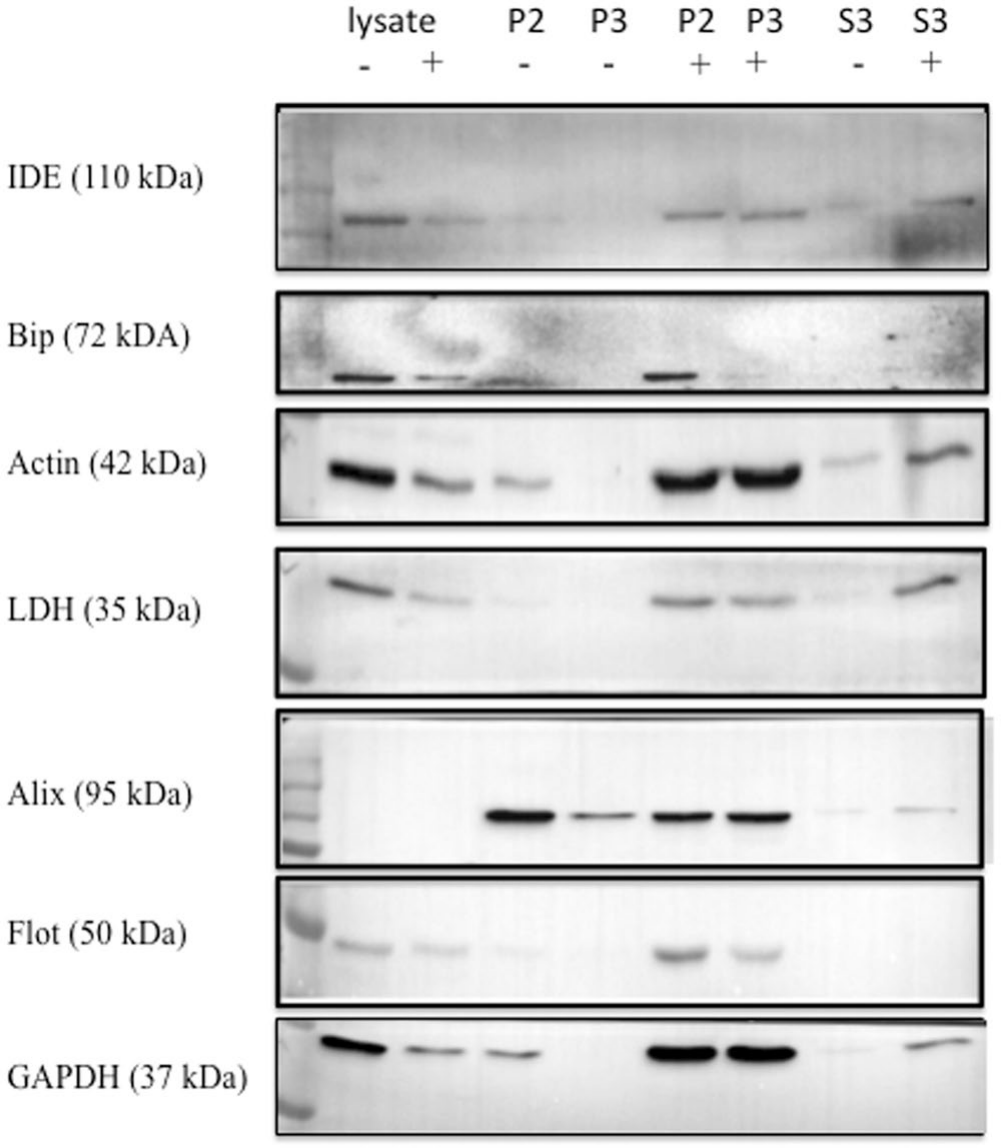
IDE and other cytosolic proteins released into media are partially associated with exosomes. BV-2 cells were cultured in DMEM media with 10% FBS for 24 h in the presence (+) or absence (−) of 5 µM lovastatin. The cell free conditioned media was centrifuged at 12,000 × *g* for 30 min. at 4 °C to yield the P2 pellet fraction. The supernatant from the P2 fraction was centrifuged at 100,000 × *g* to pellet exosomes (P3). The P2 and P3 pellets were resuspended in 100 µL of PBS and along with the supernatant (S3) and cell lysates were analyzed for the indicated proteins by Western blot analysis. In order to analyze all of the markers two SDS-PAGE gels were run. After transfer to a PVDF membrane, the membrane was cut into strips using molecular weight standards as a guide. The top part of the first membrane was cut just below the 100 KDa molecular weight marker and used for the IDE Western blot, a second cut was made just above the 50 KDa molecular weight marker and was used for Bip. Another cut was made at the 37 KDa molecular weight marker and this piece was used for the actin Western blot. The piece below the 37 KDa molecular weight marker was used for the LDH Western blot. The PVDF membrane from the second gel was cut just below the 75 KDa molecular weight marker and the top piece used for the Alix Western blot. A second cut was made at the 37 KDa molecular weight marker and this middle piece used for the Flotillin Western blot, while the lower piece of the PVDF membrane was used for the GAPDH Western blot. The entire blot strips are shown.

Tamboli *et al*.^16^ reported that a portion of the IDE in conditioned media from BV-2 cells was associated with exosomes. We thus questioned whether the proteins released from BV-2 cells were associated with exosomes and whether statin stimulated the release of exosome-associated IDE. Exosomes were isolated from conditioned media by differential centrifugation as described by Bulloj *et al*.^6^. The exosome-containing fraction, identified by the presence of Flotillin and Alix (ALG-2-interacting protein X) and the absence of Bip (binding immunoglobulin protein, also known as 78 kDa glucose-regulated protein or heat shock 70 kDa protein-5) contained IDE in agreement with the study of Tamboli *et al*.^16^ (Fig. 4). However, the exosome-enriched fraction also contained LDH, GAPDH, and actin. Further, the exosome fraction from lovastatin treated cells had higher levels of these proteins consistent with lovastatin increasing protein release via exosomes into the media. However, the amount of IDE in the exosome enriched fraction is less than 1% of the total IDE released by the cells, or less than 0.01% of total cellular IDE. This can be contrasted with the known exosomal protein Flotillin, which was present in the exosomal fraction at greater than 95% of the total cellular protein.

### Release from Neuro2a Cells

Lastly we tested for IDE secretion from Neuro2a cells as reported by Bulloj *et al*.^6^. When we grew these cells in 50% DMEM/50% Opti-MEM media containing 5% FBS as used in the study of Bulloj *et al*.^6^, the amount of IDE or LDH in the media after 18 h was barely detectable and too low to quantitate (Supplementary Fig. S6), even after concentrating the conditioned media 10 fold. In the absence of serum, the levels of IDE and LDH in the media after 18 h were again too low to measure. In addition, we isolated the exosome fraction from the conditioned media by high-speed centrifugation, but could not detect any IDE or LDH in this exosome enriched fraction.

## Discussion

In order to study IDE release from cells, we adopted protocols that would minimize or eliminate any perturbations that might induce cell dysfunction. Thus unless noted experiments were conducted in media containing FBS to stabilize the cells, and prior to measuring IDE release, media were carefully changed without disrupting cells. In addition, we either took small aliquots of the conditioned media while cells were in a 5% CO_2_ incubator or set up separate wells for time course studies so cells were not perturbed. We employed sensitive Western blot procedures so that concentration or immunoprecipitation of conditioned media was not required to detect IDE (or LDH), and total cellular IDE (or LDH) content was determined by gently washing cells on culture plates and then lysing them directly on the plate. As noted the Western blots for both IDE and LDH exhibited similar sensitivities so they could be directly compared from the same samples. These precautions should have minimized any induced cell damage during the experiment, and this was evidenced by the absence of detectable cell death as measured by the trypan blue exclusion assay and consistent with the low levels of IDE detected in the media.

The results of our analysis show that only a very small amount of IDE is released from several different cell types over an 8–24 h period, generally under 1% of total cellular enzyme, which is less than the 3–10% estimated by Zhao *et al*.^12^. Most importantly, we find that the same amount of the known cytosolic protein LDH (lactate dehydrogenase) is released with the same kinetic profile. LDH release from cells is an established marker for cellular cytotoxicity and cytolysis^22^, and its parallel release indicates that IDE in the media arises from a loss of cell integrity, not specific secretion. Thus our data question the secretion of IDE, at least in the three different cell lines we studied. Other studies report a release of IDE into the conditioned media in the absence of detectable LDH^6,11,23^. The reason for this difference is unclear but could be attributable to the differences in sensitivities of the methods used to detect IDE and LDH coupled with the low levels that are found in conditioned media. In any case, our results indicate that the bulk of released IDE seen in these studies resulted from cell disruption.

In agreement with the studies of Tamboli *et al*.^16^, we find IDE in the exosome fraction released by BV-2 cells. However, we also find LDH, GAPDH, and actin in the exosome fraction. As noted, the amount of IDE in the exosome enriched fraction is less than 1% of the total IDE found in the conditioned media or 0.01% of total cellular IDE. Thus release of IDE into the media through exosomes does not appear to be either a significant or a unique secretion mechanism. This is in contrast to the study of Bulloj *et al*.^6^, where it was reported that ~50% of the IDE in conditioned media from N2a cells was associated with exosomes. In our hands we could barely detect IDE in the conditioned media of N2a cells, and as expected, none in the exosome fraction. The reason for this difference is unclear.

We note that we have not tested all cultured cell types used for IDE secretion studies by other groups. For example, SV40-transformed murine hepatocytes and HeLa cells were used by Zhao *et al*., and we have not attempted to replicate these results. However, these studies used expressed recombinant IDE whereas our studies used endogenously expressed IDE exclusively. Nevertheless, the lack of specific IDE release from several cell type reported here makes a strong case for the absence of specific secretion.

We are left to conclude that the small amount of IDE found in conditioned media is not attributable to a specific secretion mechanism, but rather a general phenomenon associated with a loss of cell integrity. A possible mechanism is cellular dysfunction, perhaps cell death, where a small percentage of cells become leaky or lyse. This mechanism is consistent with the increase in IDE release induced by lovastatin, which may affect membrane integrity by altering cholesterol levels^24^. We hope that issues raised by this study encourage reexamination of IDE secretion and that careful quantification resolves any discrepancies in results.

If cultured cells release only very small amounts of IDE through the loss of cellular integrity, then the question arises as to the significance of extracellular IDE. That is, does IDE function as both an intracellular and extracellular enzyme in intact tissues as suggested in a number of studies^13,16,25–28^ or does it act strictly as an intracellular enzyme? This question is particularly important regarding the site of Aβ and insulin catabolism by IDE, where it has been suggested that an extracellular pool of IDE participates in the degradation these two important substrates^6,10,11,13,16,23,29–32^. However, Leissring has recently presented evidence for intracellular degradation of insulin^33^. The studies reported here do not support a significant secretion mechanism for IDE and therefore indicate that the existence and relevance of any proposed extracellular pool of IDE must be reevaluated. It is possible that nearly all degradation of Aβ and insulin by IDE occurs in one or more intracellular compartments, most likely the endosomal system^4,34–36^. We recently showed that phosphoinositols can serve as intracellular signals for IDE localization to endosomes^7^ adding support to the hypothesis that the endosomal pool of IDE might represent the major site of Aβ and insulin catabolism.

Of interest is the finding of IDE in cerebral spinal fluid^9,11^ and blood plasma^16,37^. The source of these pools of IDE has not been established nor has the activity or physiological relevance been demonstrated. Does the IDE in CSF represent a unique secreted pool of IDE or as indicated in our cellular studies is it derived from cells that have lost their cellular integrity? Going forward it will be important to better characterize these IDE pools and determine the source of the released enzyme and their physiological relevance to fully address the question of extracellular peptide degradation by IDE.

## Methods

### Antisera

The following antisera were used in these studies: anti-IDE antibodies 9B12 (Abcam) and 4020^38^, anti-LDH antibody Ab 52488 (Abcam), monoclonal anti-GAPDH (6C5) (Santa Cruz), rabbit antibody against the human pitrilysin sequence ^753^AEMTDIKPILRKLPRIKK (Bethyl Lab)^38^, monoclonal anti-Bip/GRP 78 (BD Transduction Laboratories), monoclonal anti-β-actin (C4) (Santa Cruz), monoclonal anti-Alix (1A12) (Santa Cruz), and monoclonal anti-flotillin-1 (BD Transduction Laboratories). Fetal bovine serum was purchased from Atlanta Biologicals. Two different anti-IDE antibodies, 9B12 and 4020, were used because the 9B12 monoclonal antibody, raised against human erythrocyte IDE, did not react very well with rodent IDE (BV2 and Neuro2a cells) in Western blots but did react well with human IDE in Western blots (HEK293 cells; Supplementary Fig. S7). The 4020 polyclonal antibody, rasied against rat IDE, reacted well in Western blots with rodent IDE ((BV-2, Neuro2a cells) but not well with human IDE (HEK293 cells). IDE release was calculated from data obtained with one or the other of these antibodies.

### Cell Culture and Protein Measurements

HEK293 cells (ATCC) were grown overnight in 10 cm dishes at 37 °C with 5% CO_2_ to ~75% confluence in 10 ml DMEM media (Gibco by Life technologies) containing 10% FBS. The culture media was carefully removed and 10 ml of fresh media was added. At the desired time points the plate was briefly removed from the incubator and a 0.25 ml aliquot of the media taken for analysis of secreted IDE. At the end of the incubation period the cells were washed with 10 ml of cold PBS, and 4 ml of RIPA buffer was added. After a 5 min incubation, the RIPA buffer was collected and sonicated for 20 sec with a 40% pulse using a Branson Digital Sonifier. The sonicated solution was centrifuged for 10 min at 20,000 g and used for measuring total cellular IDE and LDH. In preliminary experiments, separate cultures were used for each time point. This yielded essentially the same results, but with more scatter in the data.

Secreted IDE and LDH were measured by SDS-PAGE, running 50 µL of each sample on a 10% polyacrylamide gel. IDE was measuring using anti-IDE antibody 9B12 for HEK293 cells or anti-IDE antibody 4020 for BV-2 and Neuro2a cells, while LDH was measured using anti-LDH antibody 52488. Total cellular IDE and LDH were measured on the same gel using a 2.5 or 5.0 µL aliquot of the RIPA buffer homogenate. Bands were visualized using SuperSignal West Pico (Thermo Scientific) or SuperSignal West Femto (Thermo Scientific), scanned on a ChemiDoc MP System (BioRad), and analyzed with Image Lab software (Bio-Rad Laboratories, Inc.). Linearity of Western blot response was verified using recombinant purified IDE (Supplementary Fig. S8), and scans were taken at multiple exposure times to insure linearity. The sensitivity of these assays was very similar, with the LDH assay being slightly more sensitive than the IDE assay.

The percentage of IDE or LDH in the conditioned media was calculated as given in equation (1).

1 $$ \% \text{CM}=({\rm{TCM}}/({\rm{TCL}}+{\rm{TCM}}))\times 100$$

where %CM is the percentage of enzyme in the conditioned media, TCM is the total scanned Western blot density from conditioned media, and TCL is the total scanned Western blot density from the cell lysate.

BV-2 cells, developed by Blasi *et al*.^39^ (obtained from Dr. Elisabetta Blasi, University of Perugia), were grown overnight at 37 °C with 5% CO_2_ to ~50% confluency in 60 mm dishes containing 4 ml of DMEM media (GibCo by Life technologies) and 10% FBS. The next day the media was changed to DMEM with N2 supplemented media (GibCo by Life technologies) with and without 10% FBS or RPMI 1640 media (GibCo by Life technologies). Following overnight growth the media was changed and cells were incubated for 0, 4.5, 9, and 18 h. At each time point an aliquot of the conditioned media was carefully removed and centrifuged at 2,500 × *g* for 5 min. to remove cell debris. This conditioned media was used to assess the presence of IDE and LDH while a cell lysate was prepared as described above.

Neuro2a cells (ATCC) were grown in 50% DMEM/50% Opti-MEM media containing 5% FBS, and samples for secreted IDE and LDH were taken and analyzed as described for the other cell types.

### Lovastatin treatment

BV-2 cells grown in RPMI were treated with 5 µM lovastatin (Axxora, Enzo Life Science) for 24 h. The conditioned media was collected and assayed for the presence of IDE, LDH, GAPDH, and pitrilysin as noted above.

### Cell viability assays

For trypan blue analysis, cells were seeded in quadruplicate at ~50% confluency into 6-well plates, cultured in a 5% CO_2_ incubator at 37 °C for 24 h. In some cases lovastatin was added to a final concentration of 5 µM and cells were incubated for an additional 24 h. After the incubation period cells were treated with 1/10 of the original volume of 0.5% trypsin/EDTA for ~5 min. The detached cells were then diluted ten fold with a 0.4% trypan blue solution prepared in PBS. Following an ~5 min incubation period cells were counted using a TC10™ Automated cell counter, (Bio-Rad, Inc.). Alternatively the trypsinized cells (~100 cells) were manually counted and scored for the number of cells that had taken up trypan blue. Both methods produced similar results.

Cell metabolism was measured with the MTS assay using the CellTiter 96^®^ AQ_ueous_ One Solution Cell Proliferation Assay kit (Promega) according to the manufacturer’s instructions. This assay measures the activity of NADPH dependent dehydrogenases. Cells at ~50% confluency were seeded in quadruplicate in a 24-well plate and cultured as described above. The absorbance at 490 nm was measured with a microplate reader (SpectraMax M5, Molecular Devices).

### Isolation and Analysis of Exosomes

Exosomes were isolated as described by Bulloj *et al*.^6^. BV-2 cells were grown overnight at 37 °C with 5% CO_2_ to ~50% confluency in 7 ml of DMEM media containing 10% FBS. The next day the media was replaced with fresh media or with fresh media containing 5 µM lovastatin and incubated for 24 h. The conditioned media was then collected, centrifuged at 800 × *g* for 10 min at 4 °C to pellet the cells. The cell free conditioned media from 6 plates was combined to yield ~40 ml which was centrifuged at 12,000 × *g* for 30 min. at 4 °C to produce a pellet (P2). The supernatant from the P2 fraction was centrifuged at 100,000 × *g* for 2 h at 4 °C to pellet exosomes (P3). The pellets (P2, P3) were resuspended in 100 µl of PBS. These fractions as well as the supernatant (S3) were analyzed by Western blots using the appropriate antisera. Scans were taken for multiple exposure times to establish linearity.

## Electronic supplementary material

Supplementary Information

Dataset 1
