# Supplementary material for: Insulin-degrading enzyme is not secreted from cultured cells
Source: Sci Rep. 2018 Feb 5;8:2335. doi: 10.1038/s41598-018-20597-6 (PMC5799172; doi:10.1038/s41598-018-20597-6)
Supplement: Supplementary file 1 — Supplementary Information [file 41598_2018_20597_MOESM1_ESM.pdf]

Insulin-degrading enzyme is not secreted from cultured cells

Eun Suk Song, David W. Rodgers* and Louis B. Hersh*

Department of Molecular and Cellular Biochemistry and the Center for Structural Biology, University of Kentucky, Lexington, KY 40536

*To whom correspondence may be addressed. Email: david.rodgers@uky.edu or lhersh@uky.edu

Supplemental Information

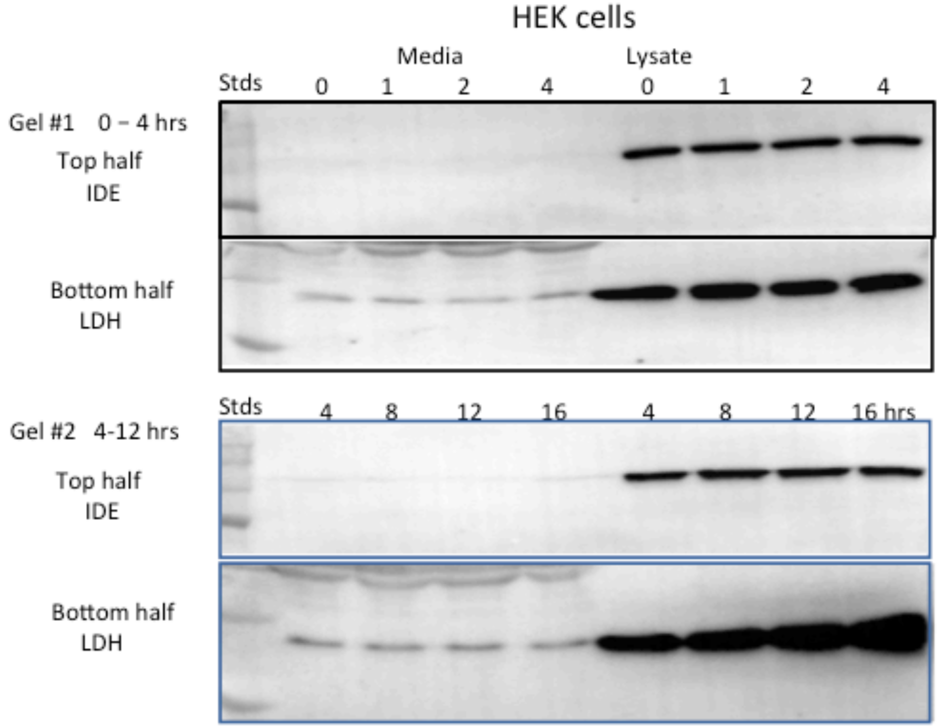

Figure S1. Example Western blot of IDE release time course from HEK cells. Aliquots of the culture supernatant (50 µL) or cell lysate (5 µL) from HEK-293 cells grown in DMEM containing 10% FBS were taken at the indicated times and subjected to Western blot analysis for IDE (top) or LDH (bottom). In order to analyze all of the time points two gels were run, the first contained the 0, 1, 2, and 4 hr samples (culture supernatant and cell lysate), while the second contained the 4, 6, 8, and 12 hr samples. Proteins were transferred to a PVDF membrane and cut at the 75KDa molecular weight marker. The upper portion of the membrane was used for the IDE Western blot, while the lower half was used for the LDH Western blot. Western blot densities were quantified using a ChemiDoc MP System with the % of IDE or LDH in the conditioned media determined from the same gel, and used to generate the curves shown in Fig. 1. The entire blot strips analyzed are shown.

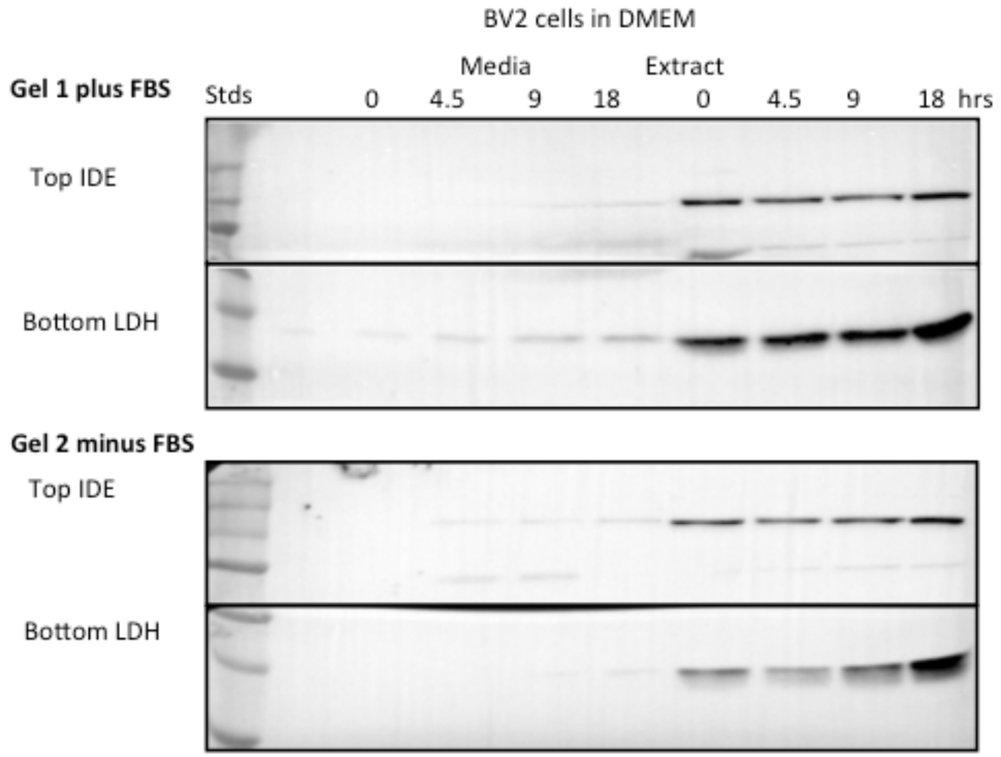

Figure S2. Example Western blot of time course of IDE release from BV-2 cells grown in DMEM. Aliquots of the culture supernatant (50 µL) or cell lysate (5 µL) from BV-2 cells grown in DMEM in the presence (top) or absence (bottom) of 10% FBS were collected at the indicated times and subjected to Western blot analysis for IDE or LDH. Two gels were run, one for the samples in the presence of FBS and the other in the absence of FBS. Following transfer to PVDF membrane the PVDF membrane was cut in half at the 75KDa marker and analyzed as in Fig. S1. The data obtained were used to generate the curves shown in the top panel of Fig. 2. The entire blot strips are shown.

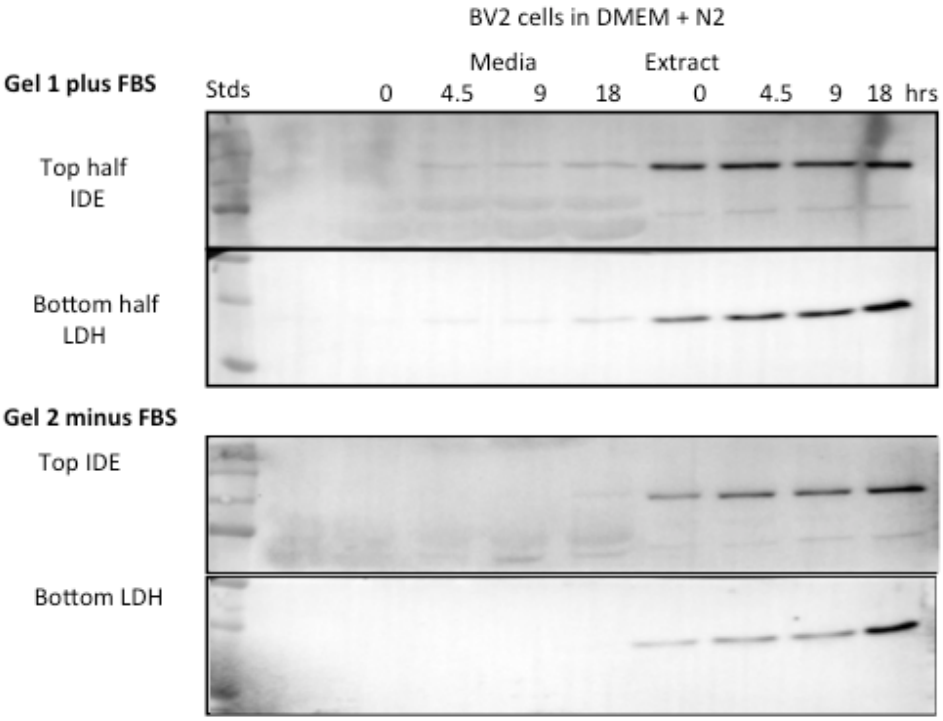

Figure S3. Example Western blot of time course of IDE release from BV-2 cells grown in DMEM plus N2 supplement. Aliquots of the culture supernatant (50 µL) or cell lysate (5 µL) from BV-2 cells grown in DMEM plus N2 supplement in the presence (top) or absence (bottom) of 10% FBS were collected at the indicated times and subjected to Western blot analysis for IDE or LDH as described in Figure S2. The Western blot densities, obtained were used to generate the curves shown in the middle panel of Fig. 2. The entire blot strips are shown.

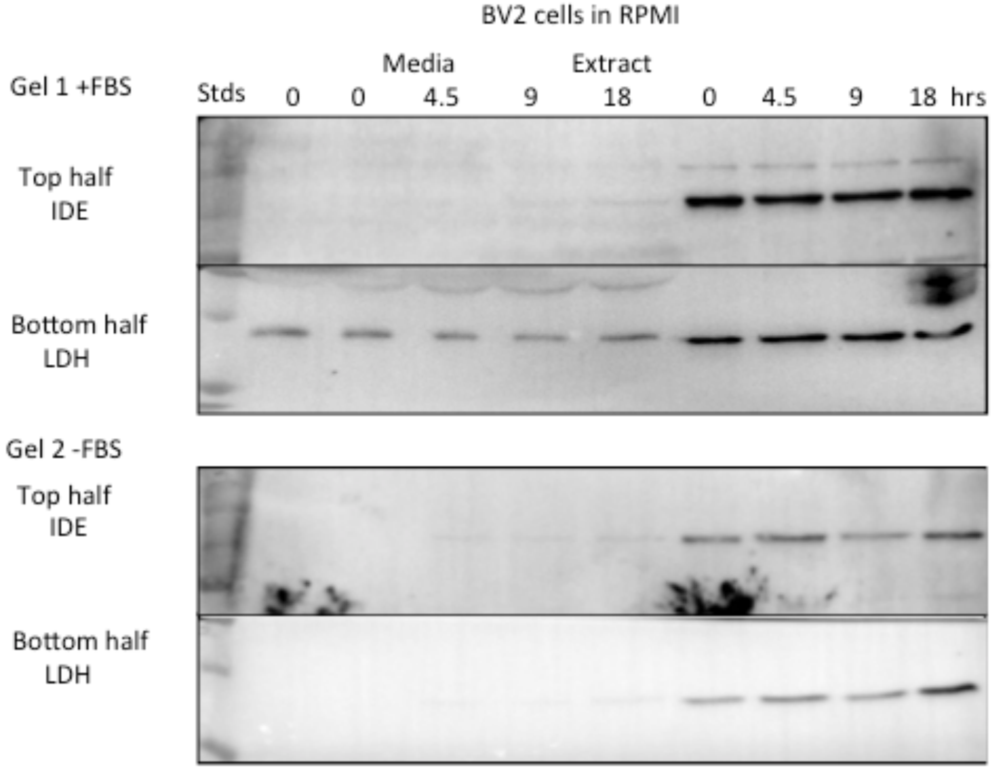

Figure S4. Example Western blot of time course of IDE release from BV-2 cells grown in RPMI. Aliquots of the culture supernatant (50 µL) or cell lysate (5 µL) from BV-2 cells grown in RPMI in the presence (top) or absence (bottom) of 10% FBS were collected at the indicated times and subjected to Western blot analysis for IDE or LDH as described in Fig. S2. The Western blot densities, obtained were used to generate the curves shown in the bottom panel of Fig. 2. The entire blot strips are shown.

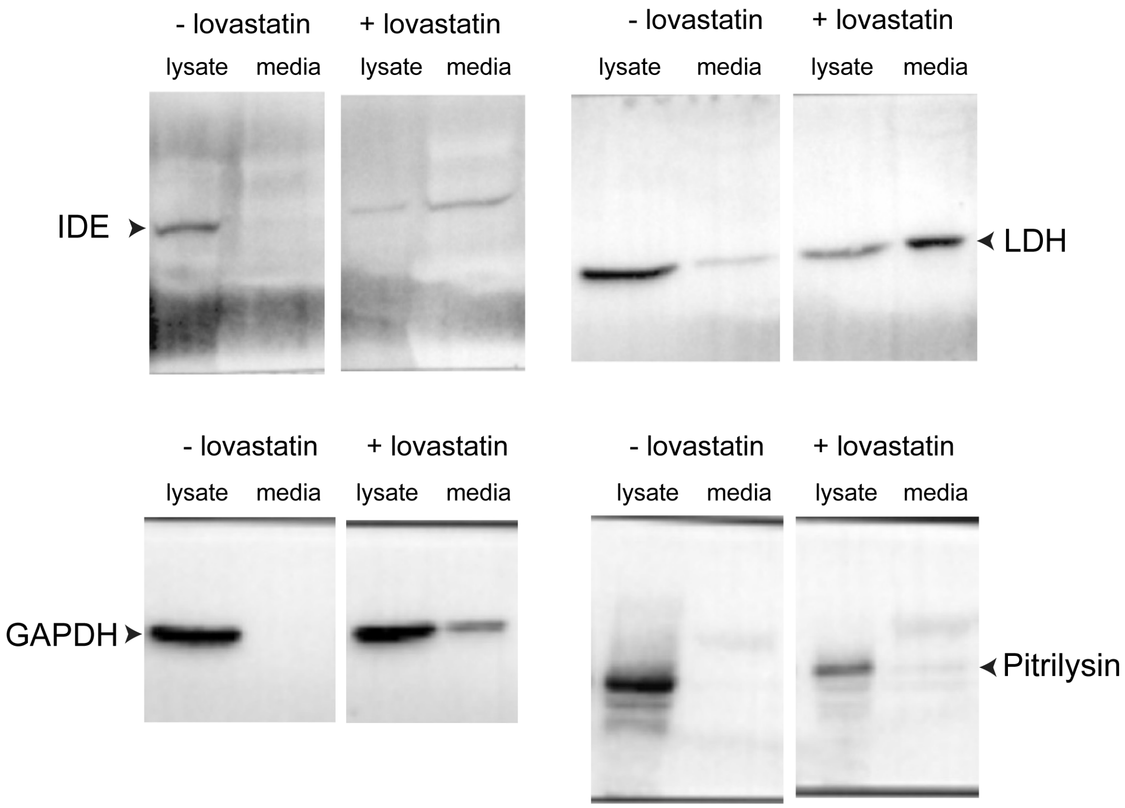

Figure S5. Example Western blot for Lovastatin induced release from BV-2 cells. Aliquots of the culture supernatant (50 µL) or cell lysate (5 µL) from BV-2 cells grown in DMEM media with 10% FBS in the presence (+) or absence (-) of 5 µM lovastatin. Samples were subject to Western blotting with appropriate antibodies (IDE, LDH, GAPDH, Pitrilysin as indicated) as described in the Methods section and Fig. S2. Western blot intensities were used to generate the histogram shown in Fig. 3.

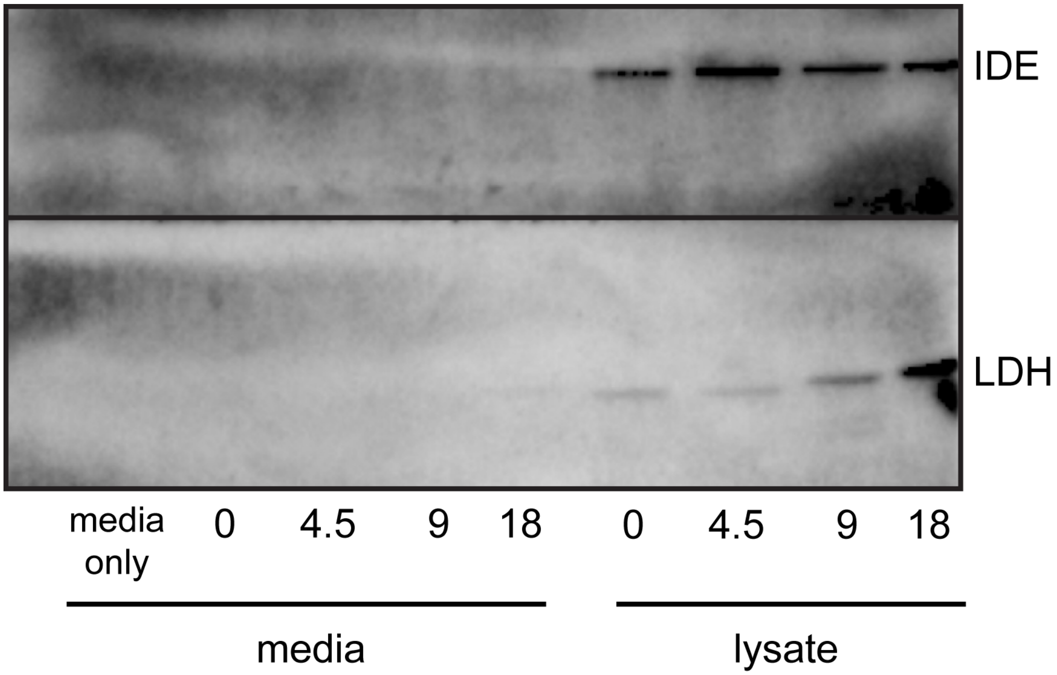

Figure S6. Lack of IDE and LDH release from Neuro2a calls. Western blot of conditioned media and cell lysate from Neuro2a cells cultured in 50% DMEM/50% Opti-MEM media containing 5% FBS. Aliquots of conditioned culture media (50 µL) or cell lysate (5 µL) were taken at the indicated times (h) and analyzed as described in the methods section. No IDE could be detected in the media, and LDH in the media was just detectable at the longest time point.

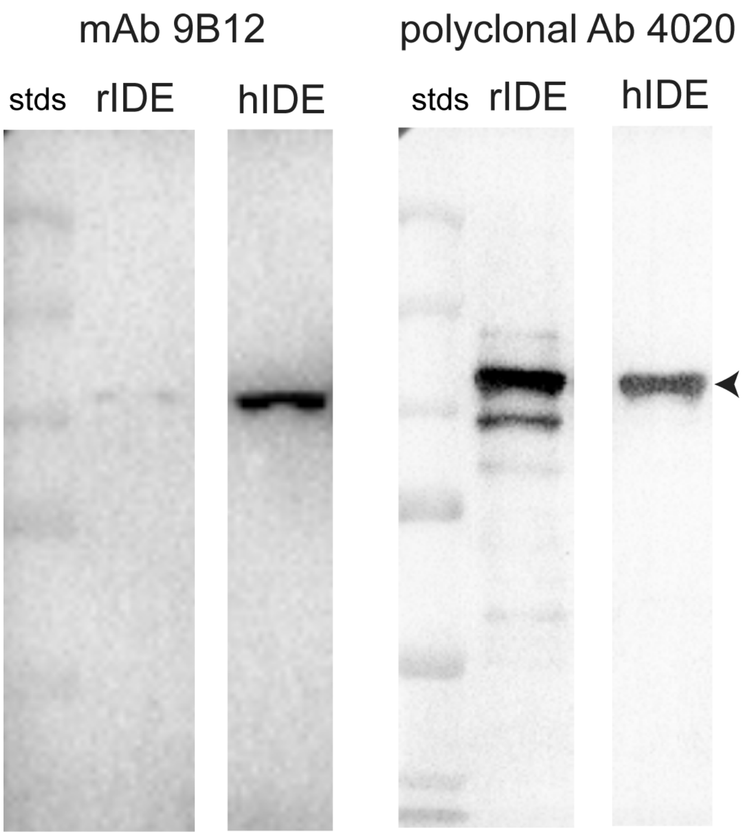

Figure S7. Western blots showing differences in IDE antibodies. Purified rat IDE (rIDE) or human IDE (hIDE) were loaded at 0.5 µg levels and blotted using either mAb 9B12 (raised against human erythrocyte IDE) or rabbit anti-IDE 4020 (raised against intact rat IDE) as described in the Methods section. It is apparent that mAb 9B12 reacts poorly with rIDE but well with hIDE (arrowhead indicates IDE position). In contrast, polyclonal Ab 4020 reacts more strongly with rIDE than hIDE.

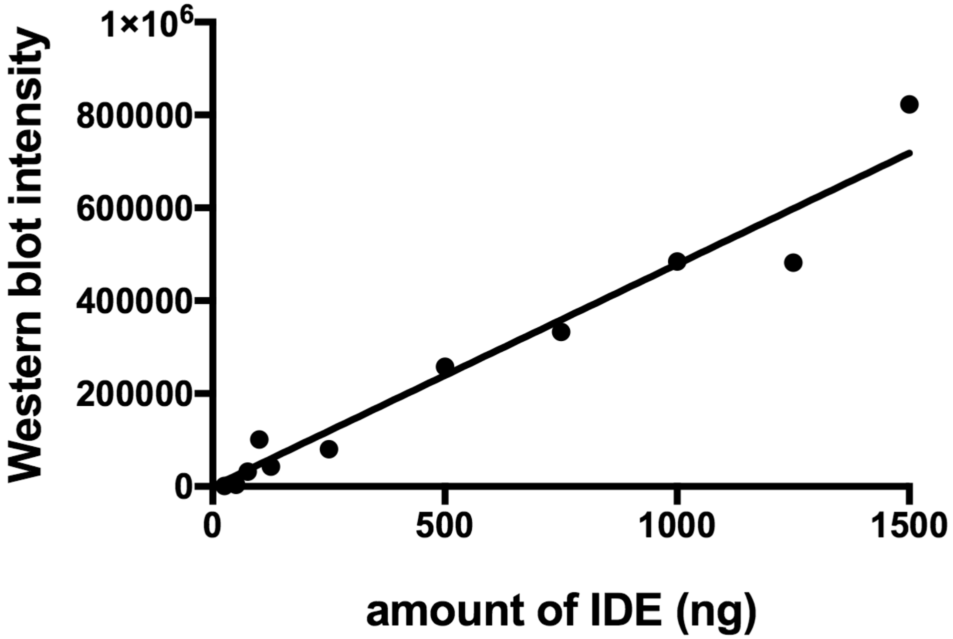

Figure S8. Linearity of Western blot quantification for IDE. The indicated amounts of purified rat IDE were subjected to Western blot analysis and quantified as described in the Methods section. The line shows a linear regression fit (GraphPad Prism), which demonstrates continued linearity at high IDE loading (overall R-squared value of 0.9563).

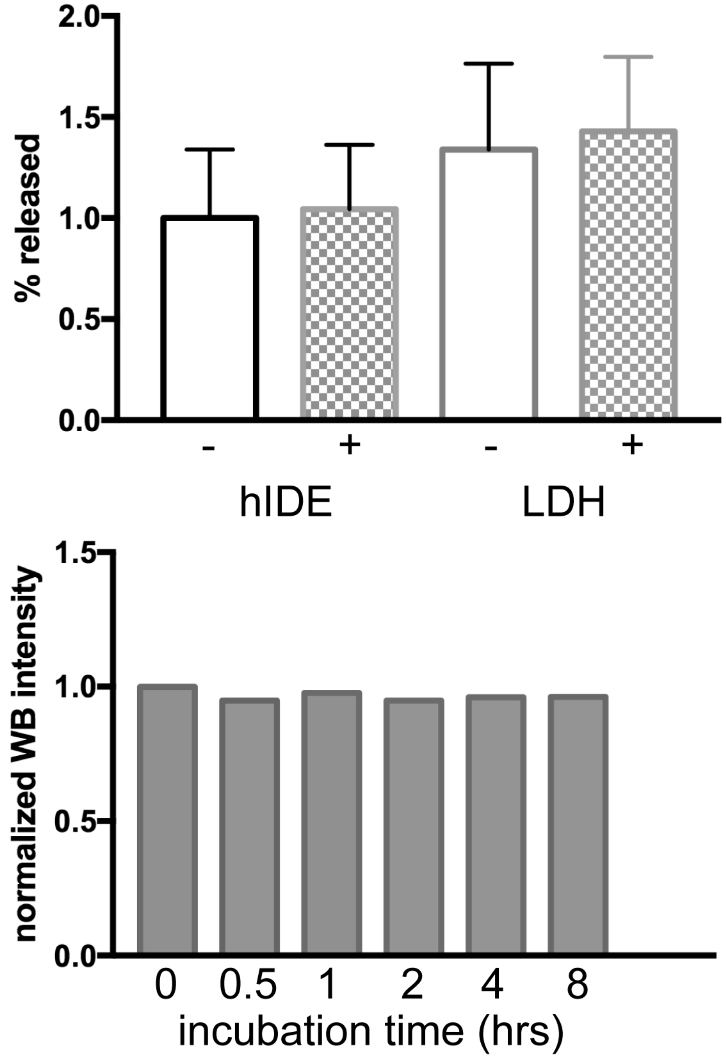

Figure S9. Stability of IDE and LDH in conditioned media. In the upper panel, percent release of IDE and LDH from HEK-293 cells in the presence (+) or absence (-) of a protease inhibitor cocktail containing AEBSF, aprotinin, bestatin, E-64, leupeptin and pepstatin A is shown. Samples were taken after a 6 h incubation and are from two independent trials. No significant differences were found due to the addition of protease inhibitors, indicating that IDE and LDH are stable in the conditioned media. In the lower panel, stability of IDE added to conditioned media is shown versus time. Recombinant rat IDE was added to conditioned media from an 18 h HEK-293 cell culture, and 200 μL samples of the media taken at the indicated times. Samples were subject to Western blot analysis and quantification as described in the Methods section. IDE remained stable in the media over the course of the incubation.

Supplementary Excel file with data for plots Figs. 1-3.
