# Supplementary material for: Insulin-degrading enzyme is not secreted from cultured cells
Source: Sci Rep. 2018 Feb 5;8:2335. doi: 10.1038/s41598-018-20597-6 (PMC5799172; doi:10.1038/s41598-018-20597-6)
Supplement: Supplementary file 2 — Dataset 1 [file 41598_2018_20597_MOESM2_ESM.docx]

**Insulin-degrading enzyme is not secreted from cultured cells**

Eun Suk Song, David W. Rodgers* and Louis B. Hersh*

Department of Molecular and Cellular Biochemistry and the Center for Structural Biology, University of Kentucky, Lexington, KY 40536

**Data from Fig. 1-3.**

**Figure 1 data**

Release of IDE and LDH from HEK-293 cells.

| time (h) | release ratio IDE data sets | | | | |
| --- | --- | --- | --- | --- | --- |
|  | 1 | 2 | 3 | 4 | 5 |
| 0 | 0 | 0 | 0 | 0 | 0 |
| 1 | 0.02544935 | 0.000742348 | 0.000742348 | 0.033150734 | - |
| 2 | 0.06355826 | 0.076102572 | 0.076102572 |  |  |
| 3 | 0.11819919 |  |  |  |  |
| 4 | 0.16938724 |  |  | 0.011461459 | - |
| 6 | 0.10325821 | 0.526672768 |  | 0.006825537 | - |
| 8 | 0.25116246 | 0.458156767 | 0.458156767 |  | 0.140788699 |

| time (h) | release ratio LDH data sets | | | | |
| --- | --- | --- | --- | --- | --- |
|  | 1 | 2 | 3 | 4 | 5 |
| 0 | 0 | 0 | 0 | 0 | 0 |
| 1 | 0.06408762 | 0.210279709 | 0.390284389 |  |  |
| 2 | 0.005054194 | 0.142430438 | 0.250724041 |  |  |
| 3 |  |  | 0.397306919 |  |  |
| 4 | 0.124986492 |  | 0.140703935 | 0.828728778 | 0.551071387 |
| 6 | 0.237427577 |  |  | 1.200002222 | 1.076658283 |

**Figure 2 data**

Top panel

| time (h) | Fig 2 top: release ratio IDE + serum data sets | | | |
| --- | --- | --- | --- | --- |
|  | 1 | 2 | 3 | 4 |
| 0 | 0 | 0 | 0 | 0 |
| 4.5 | 0.050965 |  |  |  |
| 9 | 0.620565 |  |  |  |
| 12 |  | 0.230295679 | 0.224136584 | 0.271502311 |
| 18 | 0.6150019 |  |  |  |
| 24 |  | 0.434935996 | 0.72712037 | 0.387603147 |

| time (h) | Fig 2 top: release ratio LDH + serum data sets | | | | |
| --- | --- | --- | --- | --- | --- |
|  | 1 | 2 | 3 | 4 | 5 |
| 0 | 0 | 0 | 0 | 0 | 0 |
| 4.5 | 0.2528898 |  |  |  |  |
| 9 | 0.8638494 |  |  |  |  |
| 12 |  | 0.353540618 | 0.322254577 | 0.415351587 | 0.772886824 |
| 18 |  |  |  |  |  |
| 24 | 0.5656527 | 0.2346468 | 0.447290754 | 0.190144379 | 3.454418441 |

| time (h) | Fig 2 top: release ratio IDE - serum data sets | | |
| --- | --- | --- | --- |
|  | 1 | 2 | 3 |
| 0 | 0 | 0 | 0 |
| 4.5 | 0.7464639 |  |  |
| 9 | 0.5167939 |  |  |
| 12 |  | 0.421750321 | 1.130599 |
| 18 | 0.6787839 |  |  |
| 24 |  | 1.833561258 |  |

| time (h) | Fig 2 top: release ratio LDH - serum data sets | | |
| --- | --- | --- | --- |
|  | 1 | 2 | 3 |
| 0 | 0 | 0 | 0 |
| 4.5 | 0.025810684 |  |  |
| 9 | 0.195855861 |  |  |
| 12 |  | 0.566402716 | 0.064997626 |
| 18 | 0.074199462 | 0.80235866 | 0.415194813 |
| 24 |  |  |  |

Middle panel

| time (h) | Fig 2 middle: release ratio IDE + serum data sets | | |
| --- | --- | --- | --- |
|  | 1 | 2 | 3 |
| 0 | 0 | 0 | 0 |
| 4.5 | 0.766627759 | 0.630359857 | -0.000573889 |
| 9 | 0.867025049 | 0.363205525 | 0.002960973 |
| 12 | 2.006060585 | 0.198593098 | 0.02812517 |

| time (h) | Fig 2 middle: release ratio LDH + serum data sets | | |
| --- | --- | --- | --- |
|  | 1 | 2 | 3 |
| 0 | 0 | 0 | 0 |
| 4.5 | 0.208118 | 0.374144846 | 0.149545925 |
| 9 | 0.240893758 | 0.314430476 | 0.144499555 |
| 12 | 0.521108467 | 0.402278052 | 0.571275389 |

| time (h) | Fig 2 middle: release ratio IDE - serum data sets | | |
| --- | --- | --- | --- |
|  | 1 |  |  |
| 0 | 0.036646371 |  |  |
| 4.5 | 0.248472985 |  |  |
| 9 | 0.458452079 |  |  |
| 12 | 0.036646371 |  |  |

| time (h) | Fig 2 middle: release ratio LDH - serum data sets | | |
| --- | --- | --- | --- |
|  | 1 | 2 |  |
| 0 | 0 | 0 |  |
| 4.5 | 0.42993534 | 0.001221965 |  |
| 9 | 3.026478517 | 0.001798675 |  |
| 12 |  | 0.019134612 |  |

Bottom panel

| time (h) | Fig 2 bottom: release ratio IDE + serum data sets | | |
| --- | --- | --- | --- |
|  | 1 |  |  |
| 0 | 0 |  |  |
| 4 | 0.022006596 |  |  |
| 4.5 |  |  |  |
| 6 | 0.0465068 |  |  |
| 8 | 0.127165011 |  |  |
| 9 |  |  |  |
| 12 | 0.200555716 |  |  |
| 18 |  |  |  |

| time (h) | Fig 2 bottom: release ratio LDH + serum data sets | | |
| --- | --- | --- | --- |
|  | 1 | 2 | 3 |
| 0 | 0 | 0 | 0 |
| 4 |  |  |  |
| 4.5 | 0.303338806 | 0.689221841 | 0.303338806 |
| 6 |  |  |  |
| 8 |  |  |  |
| 9 |  | 2.29268718 |  |
| 12 |  |  |  |
| 18 | 2.248786969 | 2.266552495 | 2.248786969 |

| time (h) | Fig 2 bottom: release ratio IDE - serum data sets | | |
| --- | --- | --- | --- |
|  | 1 | 2 |  |
| 0 | 0 | 0 |  |
| 4 |  |  |  |
| 4.5 | 0.268620033 | 0.567251555 |  |
| 6 |  |  |  |
| 8 |  |  |  |
| 9 | 0.953657669 | 0.9133091 |  |
| 12 |  |  |  |
| 18 | 0.827223922 | 0.963882011 |  |

| time (h) | Fig 2 bottom: release ratio LDH - serum data sets | | |
| --- | --- | --- | --- |
|  | 1 | 2 |  |
| 0 | 0 | 0 |  |
| 4 |  |  |  |
| 4.5 | 0.280849247 | 0.040424 |  |
| 6 |  |  |  |
| 8 |  |  |  |
| 9 |  | 0.214423 |  |
| 12 |  |  |  |
| 18 | 0.3521994 | 0.346523 |  |

**Figure 3 data**

| release ratio | | | | | | | |
| --- | --- | --- | --- | --- | --- | --- | --- |
| IDE | | LDH | | GAPDH | | Pitrilysin | |
| - lova | + lova | - lova | + lova | - lova | + lova | - lova | + lova |
| 0.27181 | 32.5058 | 0.419426 | 26.84127 | 1.44436 | 11.56886 | 1.383605 | 5.476595 |
| 0.91901 | 15.65666 | -0.00656 | 22.29551 |  |  |  |  |
| 0.18177 | 13.81049 | 0.90637 | 11.30126 |  |  |  |  |
